# Caspase-8 Deficient Osteoblastic Cells Display Alterations in Non-Apoptotic Pathways

**DOI:** 10.3389/fcell.2022.794407

**Published:** 2022-03-15

**Authors:** Barbora Vesela, Michael Killinger, Kamila Rihova, Petr Benes, Eva Svandová, Adela Kratochvilová, Filip Trcka, Karel Kleparnik, Eva Matalova

**Affiliations:** ^1^ Institute of Animal Physiology and Genetics, Czech Academy of Sciences, Brno, Czechia; ^2^ Faculty of Science, Masaryk University, Brno, Czechia; ^3^ Institute of Analytical Chemistry, Czech Academy of Sciences, Brno, Czechia; ^4^ International Clinical Research Center, St. Anne’s University Hospital, Brno, Czechia; ^5^ Department of Physiology, University of Veterinary Sciences Brno, Brno, Czechia

**Keywords:** osteogenesis, bone, CRISPR/cas9, apoptosis, autophagy, MC3T3-E1

## Abstract

Caspase-8 is the key component of the receptor-mediated (extrinsic) apoptotic pathway. Immunological localization of active caspase-8 showed its presence in osteoblasts, including non-apoptotic ones. Further *in vivo* exploration of caspase-8 functions in the bone is hindered by the fact that the caspase-8 knock-out is lethal prenatally. Examinations were thus performed using individual cell populations *in vitro*. In this study, caspase-8 was eliminated by the CRISPR/cas9 technology in MC3T3-E1 cells, the most common *in vitro* model of osteoblastic populations. The aim of the work was to specify the consequences of caspase-8 deficiency on non-apoptotic pathways. The impact on the osteogenic gene expression of the osteoblastic cells along with alterations in proliferation, caspase cascades and rapamycin induced autophagy response were evaluated. Osteogenic differentiation of caspase-8 deficient cells was inhibited as these cells displayed a decreased level of mineralization and lower activity of alkaline phosphatase. Among affected osteogenic genes, based on the PCR Array, major changes were observed for *Ctsk*, as down-regulated, and *Gdf10*, as up-regulated. Other significantly down-regulated genes included those coding osteocalcin, bone morphogenetic proteins (-3, -4 and -7), collagens (-1a1, -14a1) or *Phex*. The formation of autophagosomes was not altered in rapamycin-treated caspase-8 deficient cells, but expression of some autophagy-related genes, including *Tnfsf10*, *Cxcr4*, *Dapk1* and *Igf1*, was significantly downregulated. These data provide new insight into the effects of caspase-8 on non-apoptotic osteogenic pathways.

## Introduction

Caspase-8 (Casp8), a cysteine-aspartic protease, is the major initiator of the extrinsic (receptor-mediated) apoptotic pathway (Muzio et al., 1996). Caspase-8 is commonly present in cell cytoplasm as an inactive dimer. Its activation by self-processing is induced *via* death receptors and their ligands, such as Fas and FasL (Hughes et al., 2009). After activation, caspase-8 processes down-stream executor caspases (-3, -6, -7), which cleave their substrates and ensure apoptotic progression (Opdenbosch and Lamkanfi, 2019). Apart from its typical apoptotic roles, caspase-8 was described in other physiological processes including cell proliferation (Kennedy et al., 1999), differentiation (Kang et al., 2004), and autophagy (Yu et al., 2004). Not much is known about non-apoptotic functions of caspase-8 in osteogenesis and bone-related cells.

Osteoblasts are essential for bone apposition and participate in bone remodelling. Activated caspase-8 was identified in non-apoptotic osteoblasts during bone development *in situ* (Svandova et al., 2018). The functional analysis of caspase-8 in osteoblasts *in vivo* is hindered by the fact that the caspase-8 knock-out is prenatally lethal (Varfolomeev et al., 1998) before bones are formed. The most common osteoblastic *in vitro* model is the MC3T3-E1 cell line derived from early postnatal mouse calvarias (Sudo, 1983). This cell line has been successfully used as a suitable model in the last few decades (Li et al., 2017) and is often favoured because the cells synthetize and organize collagenous matrix in a similar manner as bone osteoblasts *in vivo* (Addison et al., 2015). In MC3T3-E1 cells, caspase-8 inhibition affected the cell cycle in the case of BMP4 treatment (Mogi and Togari, 2003). Additionally, caspase-8 inhibition in fully differentiated MC3T3-E1 cells caused significant changes in the expression of osteocalcin, a major osteoblastic marker (Kratochvílová et al., 2020).

These first indications about a key role of caspase-8 in osteoblasts led to the hypothesis that there might be a broader impact of caspase-8 deficiency on the differentiation process of osteoblasts. Therefore, CRISPRed MC3T3-E1 cells were generated to achieve caspase-8 deficiency during the entire differentiation process. Along with the examination of the impact of caspase-8 deficiency on the expression of an array of osteogenic factors, cell proliferation (MTT assay) was evaluated in these cells. Additionally, the consequence of caspase-8 knock-out on the activation of executive caspases and on autophagic (rapamycin induced) pathways were investigated.

## Materials and Methods

### Samples

Mice (*Mus musculus*) of the ICR strain were studied at embryonic/prenatal (E) days E13 and E15 and perinatal/postnatal (P) stage P0. The samples were obtained *post mortem* in agreement with the recent legislation in the Czech Republic, Act No. 359/2012 Sb, where according to paragraph 3, part t) “there is no specific requirement for approvals in case when the organ/tissue samples are collected *post mortem”* (which is the case of the presented investigation). Animal treatment at the IAPG runs under the approved protocols and corresponding supervision - certification 4546/2021-MZE – 18134.

Fresh *post mortem* mouse heads were fixed in 4% buffered paraformaldehyde (PFA), then dehydrated *via* gradient ethanol solutions, treated with xylene and embedded in paraffin. The MC3T3-E1 osteoblastic cell line was purchased from the European Collection of Authenticated Cell Culture (c.n. 99072810). Cells were counted by Cellometer Auto T4 (Nexcelom).

### Derivation of *Casp8* ^−/−^ MC3T3-E1 Cells

The *Casp8* ^*−/−*^ clones of MC3T3-E1 cells were generated using the CRISPR/Cas9 approach. Guide RNA (gRNA) sequences for CRISPR/Cas9 were designed by the CRISPOR online tool (Concordet and Haeussler, 2018). Two pairs of the 25-bp forward and reverse oligonucleotides (5′-CAC​CGT​AGC​TTC​TGG​GCA​TCC​TCG​A-3′, 5′-AAA​CTC​GAG​GAT​GCC​CAG​AAG​CTA​C-3′ and 5′-CAC​CGG​CTT​TTC​CAC​ATC​AGT​CGG​T-3′, 5′-AAA​CAC​CGA​CTG​ATG​TGG​AAA​AGC​C-3′) comprising 20 bp *Casp8*-target sequences and *Bsm*BI sticky ends were annealed and inserted into the lentiCRISPR v2 plasmid (Sanjana et al., 2014). Similarly, oligonucleotides comprising the GFP-target sequence were used for the derivation of a control plasmid (Knopfová et al., 2018). All plasmids were sequenced. MC3T3-E1 cells were transfected using Lipofectamine® LTX (Life Technologies), selected under puromycin (2 μg/ml) for 2 weeks. Single-cell colonies were expanded and the absence of the caspase-8 protein was verified by immunoblotting. Genomic DNA from *Casp8* ^*−/−*^ cells was isolated using the GeneElute Mammalian Genomic DNA miniprep kit (Sigma-Aldrich). PCR primers spanning the targeted gRNA site within exon 3 were designed (5′-GTG​TTG​ACC​CAG​GTT​ACA​GCT​C-3′ and 5′-TTA​GCC​CGC​AGT​CTC​ACA​AG-3′) and short Ins/Del mutations were confirmed by Sanger sequencing of PCR products. Two independent clones with mutations in different target sequences were created (clone 2 is designated as *Casp8* ^*−/−*2^).

### Cell Culture

The culturing medium consisted of MEM Alpha (Gibco, United States), FBS (10%) and penicillin/streptomycin (1,000 U/ml, 100 μg/ml). The medium was changed every 2–4 days. For detection of caspase activation, *Casp8* ^*−/−*^ and control cells were seeded in parallel at a density of 15,000 cells per cm^2^. One the second day, cells were treated by 5 µM doxorubicin (5927, Cell Signaling) for 6 h in the case of immunofluorescence and overnight for bioluminescence detection.

For the evaluation of autophagy, *Casp8* ^*−/−*^ and control cells were seeded in parallel at a density of 10,000 cells per cm^2^. On the next day, autophagy was induced by adding of 500 nM of rapamycin (R8781, Merck) to the cells followed by 72 h of cultivation.

Differentiation was induced by culturing in differentiation medium (Kratochvílová et al., 2020). *Casp8* ^*−/−*^ and control cells were seeded in parallel at a density of 5 000 cells per cm^2^. The differentiation medium was prepared as described above but with the addition of 10 mM β-glycerolphosphate and 50 μg/ml of ascorbic acid. Cells were cultured for 21 days without passaging. After 21 days, cells reached the fully differentiated state and produced an extracellular matrix as described earlier (Choi et al., 1996).

### Immunoblotting

Cell lysis and western blot analysis was performed as described before (Pekarčíková et al., 2016). The following antibodies were used: caspase-8 (4790, Cell Signaling), cleaved caspase-8 (8592, Cell Signaling), α-tubulin (T9026, Sigma), beclin-1 (612122, BD Transduction Laboratories), LC3B (2775 Cell Signaling), anti-mouse IgG HRP-linked (7076, Cell Signaling) and anti-rabbit IgG HRP-linked (7074, Cell Signaling). Blots were developed with a standard ECL procedure using Clarity Chemiluminescent Substrate (Bio-Rad, Hercules, CA).

### RNA Isolation, cDNA, PCR Array

The cultured cells were harvested into 350 μl of RLT lysis buffer (Qiagen) with β-ME (Sigma-Aldrich). The RNA was isolated by the RNeasy Mini Kit (Qiagen). Super Script VILO (Thermo Fisher Scientific) was used for cDNA preparation. Gene expression was analysed by the RT2 Profiler PCR Array (Qiagen) which allows to detect the expression of 84 genes in one run. Autophagy-related gene expression by the Mouse Autophagy Array (PAMM-084Z) and osteogenic differentiation changes by the Mouse Osteogenesis Array (PAMM-026Z). Positive and negative controls of real-time PCR are included in the PCR Array format. Samples for PCR Arrays were isolated with DNase I treatment (Qiagen) to avoid the genomic DNA contamination.

### Proliferation Analysis

Cell proliferation was determined by using the MTT assay as described previously (Pavlatovská et al., 2020). Briefly, 5 × 10^3^ of MC3T3-E1 control and *Casp8* ^*−/−*^ cells were seeded in a 24-well plate. Four days later, the medium was replaced with a fresh one containing 10% MTT (5 mg/ml stock solution, Biotech) for 4.5 h. The supernatant was removed, and the formazan was dissolved by adding 200 μl of dimethyl sulfoxide to each well. Optical density was measured as a difference at two wavelengths (570–650 nm) using the ELISA Reader Synergy HT (Bio-tek).

### Bioluminescence

For the quantification of caspase activation in control and *Casp8* ^*−/−*^ cells, an ultrasensitive and highly selective bioluminescence method based on Caspase-Glo® assays (Promega) was used. The activity of selected caspases (Caspase-Glo® 3/7 and Caspase-Glo® 6) was registered by a PMT head with a cooled photocathode (Hamamatsu 7421–40, Japan) working in photon-counting mode. The software for data acquisition and processing is an integral part of the Hamamatsu detector. The device was described in detail earlier (Ledvina et al., 2017; Killinger et al., 2021). The bioluminescence reactions take place in eight 7 µl microvials tempered to 37°C. 10 µl of diluted cell suspension (1 × 10^5^) were transferred into 30 µl of Caspase-Glo® lysis substrate and incubated for 20 min at 37°C for signal stabilization. Then lysate was added into 7-µl microvials and the bioluminescence signal was measured. The bioluminescence photon emission was counted in an interval of 10 s. The signal of 7 µl of Caspase-Glo® lysis substrate was taken as a blank.

### Immunohistochemistry and TUNEL

The PFA fixed paraffin embedded mouse heads at stages E13, 15, and P0 were used for histological sections (5 μm) in the region of the first mandibular molar segment. N = 5.

For immunohistochemical detection, antigen retrieval was carried out in the citrate buffer (pH = 6.0) for 15 min/98°C. A primary antibody against the cleaved (activated) form of caspase-8 (8592S, Cell Signaling) was applied using a dilution 1:800. The primary antibody was further detected by a peroxidase-conjugated streptavidin-biotin system (Vectastain) and a chromogen substrate diaminobenzidine (DAB, K3466; Dako) reaction was used to identify the positive cells in brown. Cell nuclei were counterstained with hematoxylin.

For the TUNEL assay (TUNEL, S7100, Merck Millipore, United States), rehydrated histological sections were pre-treated with proteinase K 20 mg/ml for 10 min/RT. The reaction mixture (3 μl TdT enzyme, 42 μl distilled water, 105 μl reaction buffer) was incubated for 45 min at 37°C. An anti-digoxigenin-peroxidase reaction was performed for 30 min at RT and a chromogen substrate diaminobenzidine (DAB, K3466, Dako) reaction was used to identify the positive cells in brown.

### Immunocytofluorescence

For immunocytofluorescence, cells were grown on culture glass and fixed by 4% PFA. The primary antibody for cleaved caspase-3 (9664, Cell Signaling) was diluted 1:50 and applied overnight/4°C. The Alexa Fluor® 488 secondary antibody (A11034, Thermo Fisher Scientific) was diluted 1:200 and applied for 40 min/RT. The LC3B Antibody kit for Autophagy (L10382, Invitrogen) was used for LC3B detection. In this case, control and rapamycin-induced cells grown on culture glass were treated by 60 µM of chloroquine 16 h before fixation to induce artificial autophagosome accumulation. The LC3B rabbit polyclonal antibody was diluted 1:1,000, applied 1h/RT and followed by the secondary antibody as described above. The cytoskeleton was visualized by the ActinGreen™ 488 ReadyProbes™ Reagent (Thermo Fisher Scientific), nuclei were detected by the ProLong® Gold Antifade reagent with DAPI (Thermo Fisher Scientific).

### Cell Staining

For staining of alizarin red and alkaline phosphatase activity, cells were grown on cultivation glass and differentiated for 3 weeks. Differentiated cells were fixed with 4% PFA. For evaluation of mineralization, cells were stained with alizarin red for 20 min. Alkaline phosphatase activity was detected by a staining mixture containing 4 mg of naphthol AS-TR phosphate disodium salt (Sigma), 150 μl of N,N-dimethylformamide (Fluka) and 12 mg of Fast blue BB Salt hemi (zinc chloride) salt (Sigma) in 15 ml of 0.1 M Tris-HCl buffer (pH 9.6) for 10 min in the dark. For spectrophotometric measurement of the alizarin red and alkaline phosphatase activity levels in the obtained cell samples, a UV–VIS spectrophotometer (Shimadzu UV-1800 Spectrophotometer) was used. The fixed and stained cells were washed by deionized water, lysed using RLT lysis buffer and homogenized using an ultrasonic homogenizer. The absorption maxima of cell lysate after alizarin red staining was determined at 525 nm and in the case of alkaline phosphatase at 597 nm.

### Statistical Analysis

The experiments were repeated three times. The expression data of the PCR Array were statistically evaluated by the Qiagen Gene Globe (https://geneglobe.qiagen.com/us/) as recommended by the producer. Three independent biological samples were analysed for each experiment. Statistical significance was calculated by a *t*-test. The housekeeping genes included *Actb*, *B2m*, *Gapdh*, *Gusb*, and *Hsp90ab1*. Significance was determined as *p* < 0.05, and the fold regulation threshold was ±2. MTT assay data and bioluminescence results were tested on *p* < 0.05 by ANOVA. Three independent measurements were performed; samples were measured in duplicates.

## Results

### Caspase-8 is Activated in Apoptotic as Well as Non-Apoptotic Bone Cells

**FIGURE 1:**
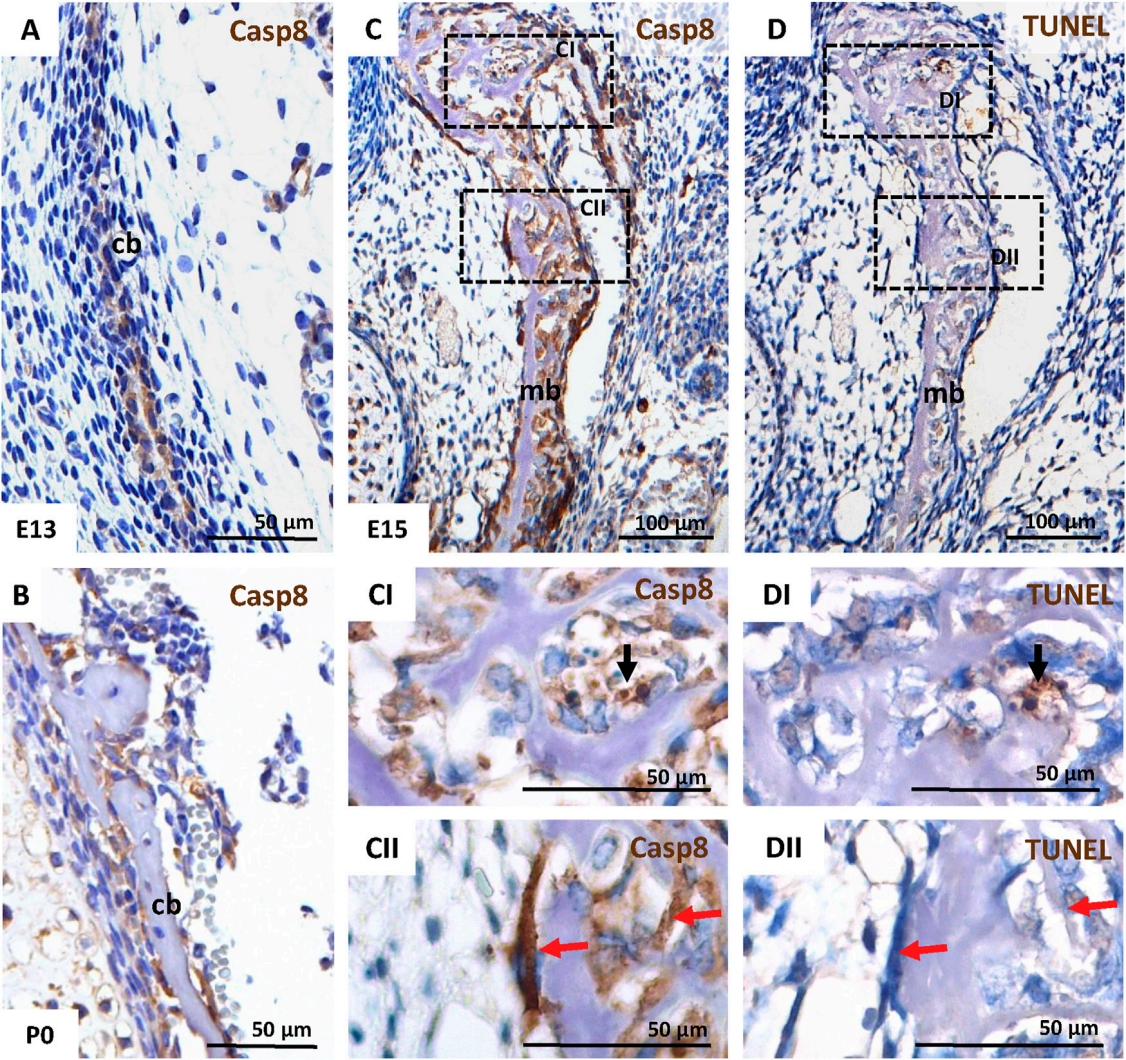
Activation of caspase-8 in the intramembranous skull bones in the mouse. IHC of caspase-8 activation in the calvarial bone at E13 **(A)**, and P0 **(B)**, and in mandibular bone at E15 **(C,CI,CII)**. Apoptotic cells in mandibular bone at E15 were detected using TUNEL assay **(D,DI,DII)**. Black arrows mark apoptotic cells; red arrows mark non-apoptotic cells in serial sections of mandibular bone. Scale bar = 100 µm for C, D; Scale-bar = 50 µm for A, B, CI, CII, DI, DII. **cb** calvarial bone, **mb** mandibular bone.

MC3T3-E1 osteoblastic cells are derived from mouse calvarial bone. IHC was used to analyse caspase-8 activation (cleaved caspase-8) during the formation of these bones *in vivo*. Activation of caspase-8 was observed in calvarial cells from an early stage of osteoid secretion by pre-osteoblasts/osteoblasts at E13 (Figure 1A), untill more advanced stages when osteocytes became entrapped in bone extracellular matrix at P0 (Figure 1B).

Furthermore, caspase-8 was activated in cells of the developing mandibular bone (another model of intramembranous ossification) at E15 (Figures 1C, CI, CII). Notably, besides a few cells with caspase-8 activation (Figure 1CI), which correlated with apoptotic cells detected *via* TUNEL staining (Figure 1DI), many non-apoptotic osteoblasts (Figure 1DII) displayed the activation of caspase-8 (Figure 1CII).

### *Casp8* ^*−/−*^ Osteoblasts Are Viable but Exhibit Decreased Proliferation

**FIGURE 2:**
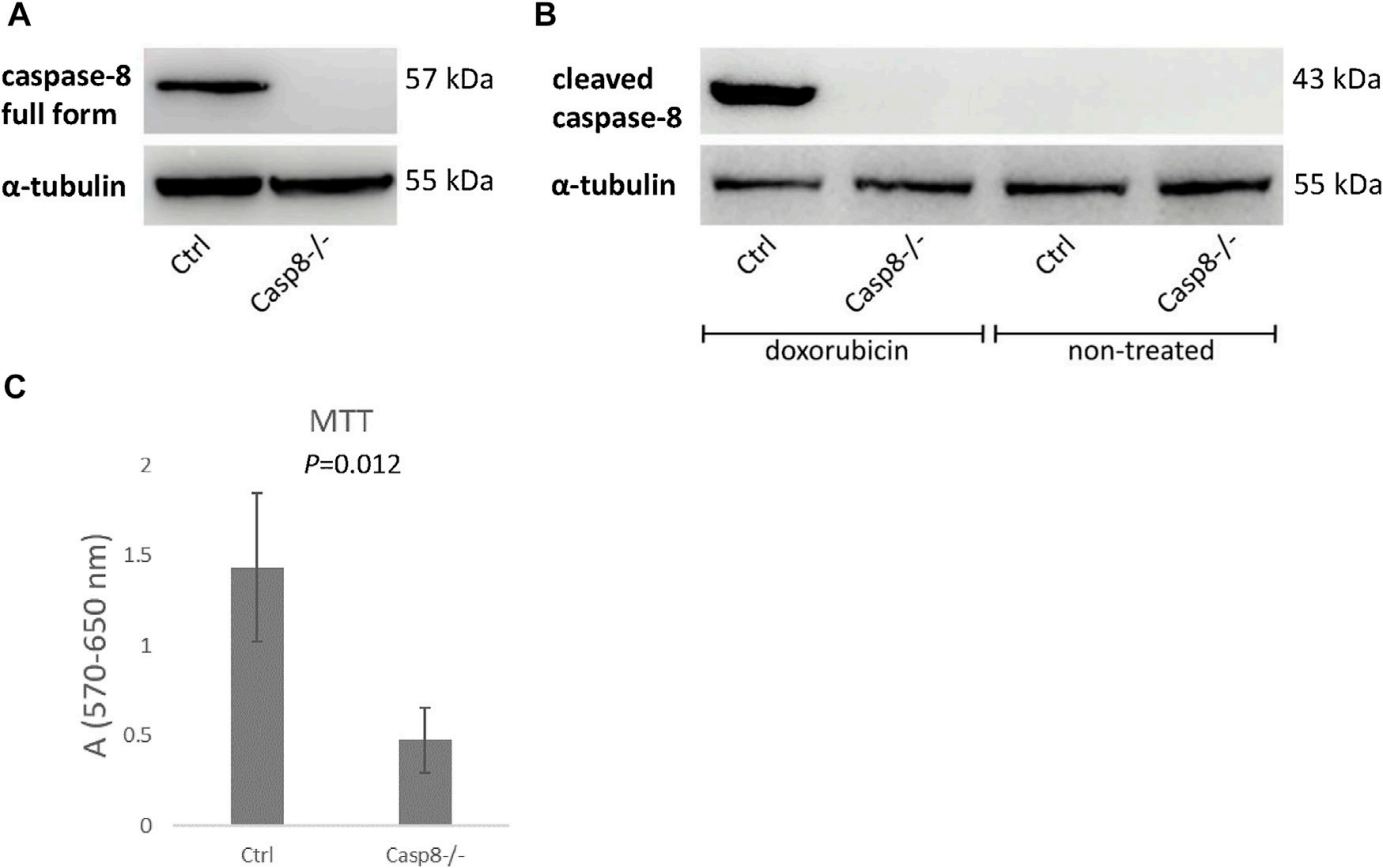
Immunoblotting of full length caspase-8 proenzyme in unstimulated *Casp8* ^*−/−*^ and control cells **(A)** and cleaved caspase-8 in doxorubicin stimulated *Casp8* ^*−/−*^ and control cells **(B)**. Cell proliferation of *Casp8* ^*−/−*^ cells compared to control cells evaluated by MTT test **(C)**.

MC3T3-E1 *Casp8* ^*−/−*^ cells were derived using the CRISPR/Cas9 approach. The absence of the caspase-8 proenzyme (full form) in unstimulated cells and cleaved caspase-8 (active form) in doxorubicin-treated cells was confirmed by immunoblotting (Figures 2A,B) (Supplementary Figures S1A,B). The CRISPR/Cas9 approach generated viable *Casp8* ^*−/−*^ osteoblastic cells, but with significantly (*p* = 0.012) decreased cell proliferation as confirmed by the MTT test (Figure 2C) (Supplementary Figure S1C).

### Caspase-8 Deficiency Affects the Activation of Effector Caspases

**FIGURE 3:**
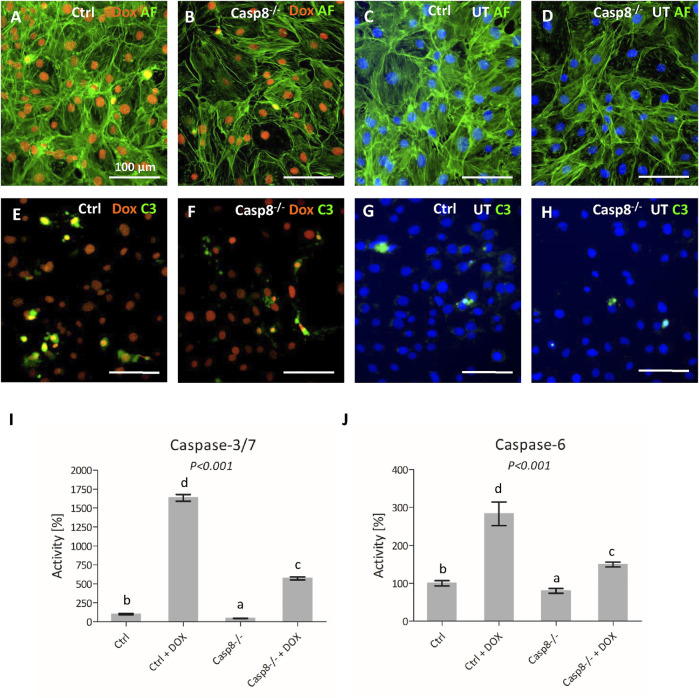
Staining of actin filaments (green) of control and *Casp8* ^*−/−*^ cells after doxorubicin treatment compared to untreated controls **(A–D)**. Activation of caspase-3 (green) in control and *Casp8* ^*−/−*^ cells after doxorubicin treatment compared to untreated controls **(E–H)**. Nuclei of doxorubicin treated samples are red, untreated samples are counterstained by DAPI (blue). Scale bar = 100 µm. Bioluminescent measurement of caspase3/7 **(I)** and caspase-6 **(J)** activity in *Casp8* ^*−/−*^ and control cells after doxorubicin treatment compared to untreated controls. Different letters indicate statistically significant differences (*p* < 0.001), as estimated using ANOVA. **Dox** doxorubicin, **UT** untreated.

To further confirm the efficiency of the *Casp8* knock-out, *Casp8* ^*−/−*^ and control cells were treated by doxorubicin, a well-known apoptotic inducer, which can activate both, mitochondrial and receptor-mediated apoptotic pathways (Xie et al., 2008; Christidi and Brunham, 2021). A fluorescent signal for doxorubicin was detected in the nuclei of treated *Casp8* ^*−/−*^ and control cells (Figures 3A,B), cells were visualized by staining of actin filaments (Figures 3A–D). Caspase-3 activity was increased in treated groups compared to untreated ones (Figures 3E–H). Among the treated cells, a higher caspase-3 activity was observed in control cells compared to caspase-8 deficient ones (Figures 3E,F).

Bioluminescence detection of effector caspase activity revealed a statistically significant decrease (*p* < 0.001) in the activation of caspases-3/7 and caspase-6 in *Casp8* ^*−/−*^ cells after apoptosis stimulation. The activity of caspase-3/7 (Figure 3I) in *Casp8* ^*−/−*^ proliferating cells was about one half of what was measured in the control ones. After the doxorubicin treatment, the control cells exhibited an enormous increase of active caspase-3/7 (almost 20 times), compared to *Casp8* ^*−/−*^ cells, where the signal was only six times higher than in untreated cells. The activity of caspase-6 (Figure 3J) was lower than caspase-3/7. The activity in *Casp8* ^*−/−*^ cells was just slightly lower than in control cells. On the contrary, after doxorubicin treatment, the signal raised almost three times in the control group, but just 1.5 times in *Casp8* ^*−/−*^ cells.

### Caspase-8 Deficiency Causes Only Minor Changes Connected With Autophagy

**FIGURE 4:**
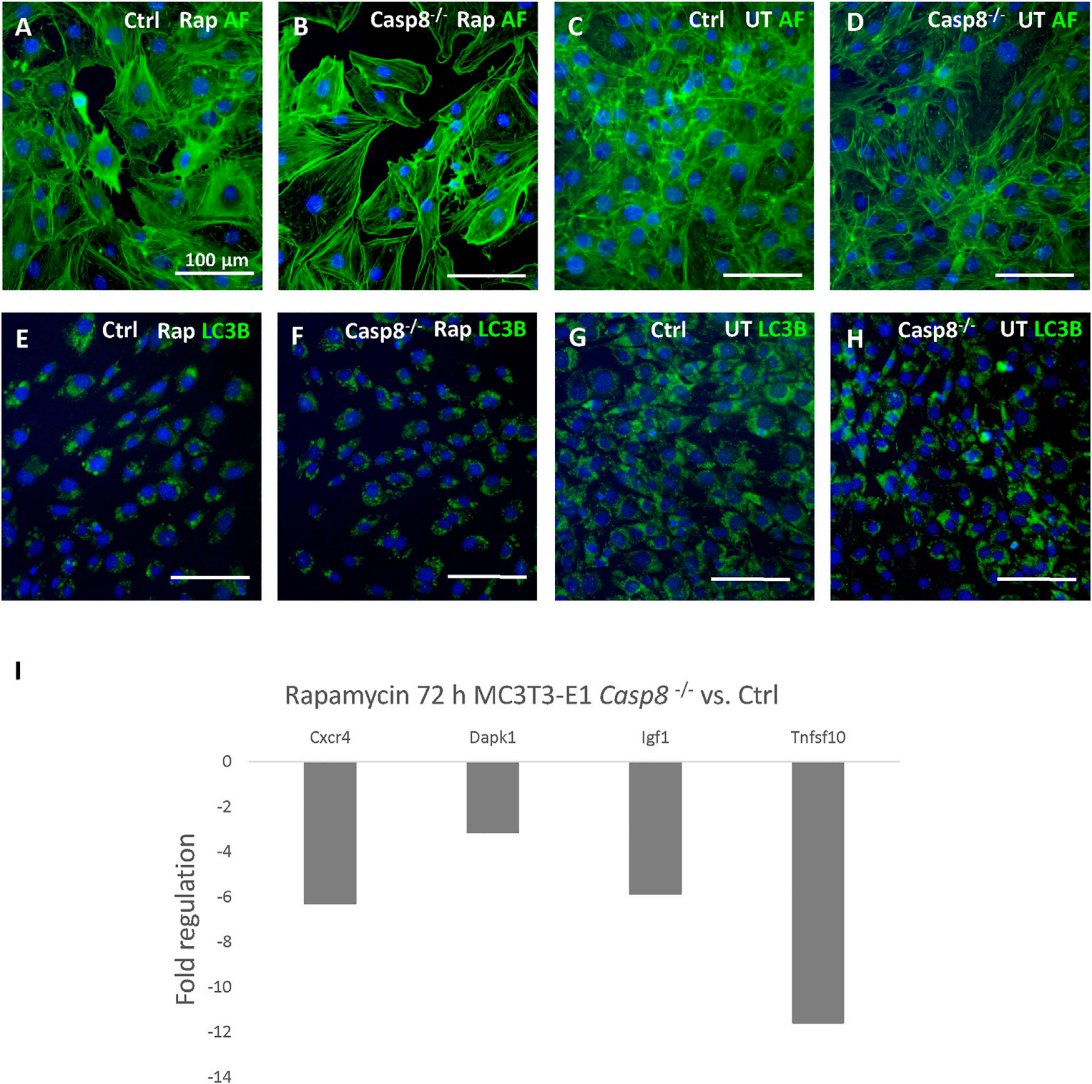
Staining of actin filaments (green) of control and *Casp8* ^*−/−*^ cells after rapamycin treatment compared to untreated controls **(A–D)**. Staining of LC3B (green) in artificially accumulated autophagosomes in control and *Casp8* ^*−/−*^ cells after rapamycin treatment compared to untreated controls **(E–H)**. Autophagosomes accumulation was induced by chloroquine. Nuclei counterstained by DAPI (blue). Scale bar = 100 µm. PCR Array analysis of autophagy-related gene expression after 72 h of rapamycin treatment in *Casp8* ^*−/−*^ compared to control cells **(I)**. **Rap** rapamycin, **UT** untreated.

*Casp8* ^*−/−*^ and control cells were induced by rapamycin. Treated cells in both groups displayed a decreased cell density (Figures 4A–D). LC3B staining of artificially accumulated autophagosomes did not display any differences between *Casp8* ^*−/−*^ and control cells (Figures 4E–H) and (Supplementary Figures S1E–H). Similarly, no differences were observed in the levels of Beclin-1 and LC3B between *Casp8* ^*−/−*^ and control cells using immunoblotting analysis (Supplementary Figure S1D). Out of 84 autophagy-related genes, the expression of four genes was significantly decreased in caspase-8 deficient cells after rapamycin treatment (Figure 4I). Among these, the strongest decrease was detected in *Tnfsf10*, gene coding for Trail (fold regulation: −11.58; *p* < 0.001). Other genes with a decreased expression included *Cxcr4* (−6.29; *p* < 0.001), *Dapk1* (−3.14; *p* = 0.048) and *Igf1* (−5.87; *p* < 0.001). All analysed genes are listed in Supplementary Figure S2.

### Caspase-8 Deficiency Affects the Expression of Osteogenic Markers

**FIGURE 5:**
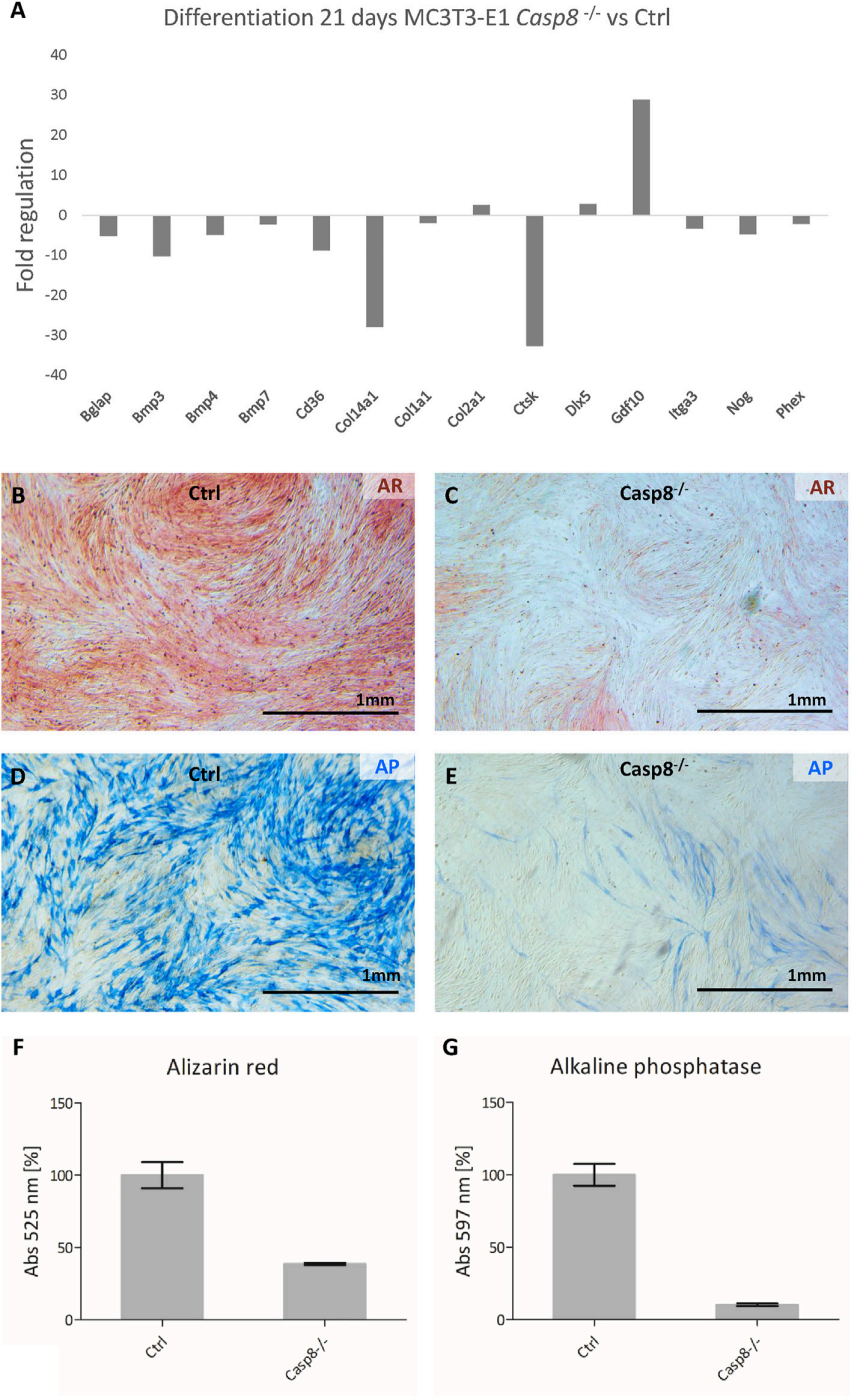
PCR Array analysis of osteogenesis-related gene expression after 21 days of differentiation in *Casp8* ^*−/−*^ compared to control cells **(A)**. Alizarin red staining of mineralization (red) in control **(B)** and *Casp8* ^*−/−*^ **(C)** differentiated cells. Alkaline phosphatase activity (blue) in control **(D)** and *Casp8* ^*−/−*^ **(E)** differentiated cells. Scale bar = 1 mm. Spectrophotometric analysis of alizarin red **(F)** and alkaline phosphatase activity **(G)** in cell lysates of stained control and *Casp8* ^*−/−*^ differentiated cells.

After 3 weeks of differentiation, osteogenic gene expression in control cells was compared to that of *Casp8* ^−/−^ cells (Figure 5A). The most prominent decrease was detected for *Ctsk*, a gene coding for cathepsin K (fold regulation: 32.7; *p* < 0.001). Other genes with a decreased expression included *Bglap*, a gene coding for osteocalcin (−5.17; *p* < 0.001), *Bmp3* (−10.23; *p* = 0.012), *Bmp4* (−4,85; *p* < 0.001), *Bmp7* (−2.25; *p* = 0.003), *Cd36* (−8.84; *p* = 0.032), *Col14a1* (−27.94; *p* = 0.002), *Itga3* (−3.34; *p* < 0.001), *Nog* (−4.74; *p* < 0.001) and *Phex* (−2.18; *p* < 0.001). Just below 2-fold regulation was expression of *Col1a1* (−1.95; *p* < 0.001). Increased expression was detected for *Col2a1* (2.59; *p* = 0.006), *Dlx5* (2.77; *p* = 0.001) and *Gdf10* (28.82; *p* < 0.001). All analysed genes are listed in Supplementary Figure S3.

Alizarin red staining showed abundant mineralization in control cells and decreased mineralization in *Casp8* ^*−/−*^ cells (Figures 5B,C), which was confirmed by the UV-VIS spectrophotometry. The absorption of *Casp8* ^*−/−*^ samples was a half level of the control samples (Figure 5F). Similarly, alkaline phosphatase activity was markedly higher in differentiated control cells than in *Casp8* ^*−/−*^ cells (Figures 5D,E). In this case, the absorption of *Casp8* ^*−/−*^ samples was decreased ten times compared to control samples (Figure 5G).

## Discussion

In this research, the non-apoptotic roles of caspase-8 in osteoblasts were investigated with a focus on the differentiation of these cells. The *in vitro* CRISPR/Cas9 approach was an alternative because of the embryonic lethality of caspase-8 knock-out mice before the onset of osteoblastic differentiation (Varfolomeev et al., 1998).

Embryonic lethality of caspase-8 deficient animals is caused by to the caspase-8/Ripk3 pathway (Kaiser et al., 2011). Whereas caspase-8 suppresses Ripk3 or Mlkl-dependent necroptosis in wild type mice, Casp8-deficient embryos lose these defence mechanisms and die from consecutive cardiac, vascular and hematopoietic defects (Varfolomeev et al., 1998; Kaiser et al., 2011; Fritsch et al., 2019). Similar molecular networks were observed in osteoblastic MC3T3-E1 cells. In TNF-α treated cells, apoptosis was induced, while a combined treatment with a caspase-8 Z-IETD-FMK inhibitor led to increased Ripk3 expression and Mlcl phosphorylation initiating necroptosis (Shi et al., 2019). Thus, deficiency of caspase-8 activation, as expected, changed the sensitivity to apoptosis and cell viability. Similar effects were also observed in MC3T3-E1 Casp8^−/−^ cells. The activation of the executive trio of caspases (3, -6 and -7) was also significantly reduced in doxorubicin-treated Casp8^−/−^ cells, as a consequence of the disruption of the caspase cascade. Notably, caspase-8 deficient osteoblasts were viable, but showed significantly decreased proliferation. Previously, caspase-8 was confirmed as a critical regulator of proliferation in murine as well as human immune cells (Chun et al., 2002; Salmena and Hakem, 2005). Nevertheless, the role of caspase-8 in osteoblast proliferation has not yet been clearly specified.

Along with apoptosis, caspase-8 was described in other modes of cell death, particularly autophagy (Yu et al., 2004). Apoptosis and autophagy are interconnected processes (Mizushima and Komatsu, 2011) and are involved in physiological development, including bone remodelling and homeostasis (Nollet et al., 2014). To evaluate autophagy response in the Casp8^−/−^ osteoblasts, the cells were treated by rapamycin, a well-known mTOR inhibitor (Jung et al., 2010). Caspase-8 deficiency did not alter the formation of autophagosomes, but decreased the expression of four autophagy-related genes with the most apparent change in Tnfsf10, the gene for Trail. Besides the traditional apoptotic function, Trail is an important autophagic player balancing autophagy and apoptosis (Sharma and Almasan, 2018). Trail-resistance in prostate cancer cells was reported to increase autophagy, and on the contrary, Trail sensitivity favoured apoptosis (Ray et al., 2005). The situation in osteoblasts is much more complex. These cells display different responses to Trail based on their differentiation status (Brunetti et al., 2013). Focused on other dysregulated genes upon autophagy stimulation in Casp8^−/−^ osteoblasts, decreased levels of Igf1 and Dapk1 are associated with attenuated autophagy (Inbal et al., 2002; Renna et al., 2013). No significant changes were revealed in the expression of important autophagy markers included in the PCR Array format (e.g., Becn1, Lc3B, Atgs) which correspond to similar protein levels of Beclin-1 and LC3B in control and caspase-8 deficient cells.

Non-apoptotic functions of caspases in cell differentiation have been discussed in general (Shalini et al., 2015; Julien and Wells, 2017). In osteoblasts, non-apoptotic activation of caspase-8 was assumed to play a role in the regulation of their differentiation (Mogi and Togari, 2003). Additionally, caspase-8 inhibition in fully differentiated MC3T3-E1 caused a decrease in the expression of osteogenic factors Bglap (gene for osteocalcin) and Phex (Kratochvílová et al., 2020). In this study, the caspase-8 deficiency in osteoblastic cells during differentiation confirmed an even stronger downregulation of Bglap and Phex. Several other osteogenesis-related genes were dysregulated in the case of caspase-8 deficiency. Notably, the most prominent down-regulation was observed for Ctsk, the gene for cathepsin K. Cathepsin K is a cysteine protease highly produced by osteoclasts involved in bone remodelling (Drake et al., 2017). Moreover, it can be produced by other cell types such as osteoblasts and osteocytes (Lotinun et al., 2019). As shown previously, an increased expression of Ctsk is connected with MC3T3-E1 cells differentiation (Kratochvílová et al., 2020). In human osteoblasts, the production of cathepsin K was described as related to collagenous matrix maintenance (Mandelin et al., 2006). A significant downregulation of Ctsk in caspase-8 deficient cells suggests a disrupted differentiation process. Similarly, lower differentiation of Casp8^−/−^ cells is supported by decreased expressions of some Bmps (Hiraki et al., 1991; Chang et al., 2009; Lavery et al., 2009; Kokabu and Rosen, 2018). This is in accordance with the significantly upregulated Gdf10 observed in caspase-8 deficient cells. Gdf10 (also called Bmp-3b) is a member of the TGFβ superfamily and is closely related to Bmp3 (Hino et al., 2004). However, Gdf10 inhibits osteoblastic differentiation (Matsumoto et al., 2012), where it supresses the expression of osteogenic factors including osteocalcin and collagen type 1. These two factors were also decreased in Casp8^−/−^ cells. Among genes downregulated in caspase-8 deficient cells, there was also Cd36. Cd36 is an important component involved in the regulation of osteoblast metabolism and its deficiency decreased the expression of osteocalcin and other osteogenic markers (Kevorkova et al., 2013). Plasma levels of osteocalcin and N-terminal propeptide of type I procollagen were significantly decreased in the CD36KO mouse. Notably, caspase-8 deficiency affected the expression of Col14a1. Collagen type 14 was described in tendons and skin where it regulates fibrillogenesis (Ansorge et al., 2009), but there is not much information about its roles in osteoblasts. Nevertheless, it is also activated in fibrillogenesis related to tooth-bone anchorage (Zvackova et al., 2017). A significant effect of caspase-8 on osteoblastic differentiation was confirmed by reduced mineralization and alkaline phosphatase activity in deficient cells. It is in accordance with lower alkaline phosphatase activity observed after general caspase inhibition in differentiated MC3T3-E1 cells (Kratochvílová et al., 2020) and after caspase-8 inhibition in BMP4-treated MC3T3-E1 cells (Mogi and Togari, 2003).

Taken together, the data presented here confirmed our hypothesis that caspase-8 has a broader impact on osteoblastic differentiation, and identified several molecules involved in this process. Additionally, caspase-8 deficiency inhibited the proliferation of osteoblastic cells. Since caspases are considered as possible targets in many current therapies including cancer or metabolic disorders (Wilson and Kumar, 2018; Kostova et al., 2021), knowledge about their pleiotropic effects is of particular importance. Despite the fact that our results were obtained using a specific cell line, MC3T3-E1 cells share much in common with *in vivo* osteoblasts (Addison et al., 2015), and the identification of the molecules and pathways affected by caspase-8 deficiency thus allow for broader extrapolations.

## Data Availability

The original contributions presented in the study are included in the article/Supplementary Material, further inquiries can be directed to the corresponding author.
