# Supplementary material for: Caspase-8 Deficient Osteoblastic Cells Display Alterations in Non-Apoptotic Pathways
Source: Front Cell Dev Biol. 2022 Mar 15;10:794407. doi: 10.3389/fcell.2022.794407 (PMC8964645; doi:10.3389/fcell.2022.794407)
Supplement: Supplementary file 1 [file DataSheet3.DOCX]

| **gene** | **fold reg.** | **P-value** |  | Igf1r | 1.10 | 0.333393 |
| --- | --- | --- | --- | --- | --- | --- |
| Acvr1 | 1.35 | 0.000650 |  | Ihh | 1.17 | 0.801161 |
| Ahsg | -1.67 | 0.238833 |  | Itga2 | -1.31 | 0.521984 |
| Alpl | 1.06 | 0.414725 |  | Itga2b | -1.30 | 0.110409 |
| Anxa5 | -1.10 | 0.248724 |  | Itga3 | -3.34 | 0.000283 |
| Bglap | -5.17 | 0.000039 |  | Itgam | -1.17 | 0.014325 |
| Bgn | 1.53 | 0.000035 |  | Itgav | -1.01 | 0.822572 |
| Bmp1 | -1.11 | 0.093553 |  | Itgb1 | -1.02 | 0.406114 |
| Bmp2 | -1.26 | 0.022793 |  | Mmp10 | -1.30 | 0.038564 |
| Bmp3 | -10.23 | 0.012607 |  | Mmp2 | 1.20 | 0.003662 |
| Bmp4 | -4.85 | 0.000097 |  | Mmp8 | -1.16 | 0.385773 |
| Bmp5 | -1.26 | 0.022793 |  | Mmp9 | -1.45 | 0.140017 |
| Bmp6 | -1.69 | 0.102515 |  | Nfkb1 | 1.07 | 0.138776 |
| Bmp7 | -2.25 | 0.002767 |  | Nog | -4.74 | 0.000295 |
| Bmpr1a | 1.19 | 0.010967 |  | Pdgfa | -1.06 | 0.325067 |
| Bmpr1b | -1.02 | 0.934317 |  | Phex | -2.18 | 0.000040 |
| Bmpr2 | -1.12 | 0.078767 |  | Runx2 | -1.00 | 0.940946 |
| Cd36 | -8.84 | 0.031930 |  | Serpinh1 | -1.31 | 0.006043 |
| Cdh11 | 1.02 | 0.430039 |  | Smad1 | 1.12 | 0.054682 |
| Chrd | -1.24 | 0.000830 |  | Smad2 | 1.10 | 0.025357 |
| Col10a1 | -1.25 | 0.234210 |  | Smad3 | -1.30 | 0.000447 |
| Col14a1 | -27.94 | 0.002948 |  | Smad4 | 1.04 | 0.217511 |
| Col1a1 | -1.95 | 0.000659 |  | Smad5 | -1.08 | 0.218417 |
| Col1a2 | -1.70 | 0.000018 |  | Sost | -1.26 | 0.022793 |
| Col2a1 | 2.59 | 0.006730 |  | Sox9 | 1.56 | 0.002159 |
| Col3a1 | 1.12 | 0.099584 |  | Sp7 | -1.35 | 0.014895 |
| Col4a1 | -1.07 | 0.102106 |  | Spp1 | -2.00 | 0.003436 |
| Col5a1 | -1.38 | 0.006250 |  | Tgfb1 | -1.90 | 0.000006 |
| Comp | 1.62 | 0.000737 |  | Tgfb2 | 1.14 | 0.106529 |
| Csf1 | -1.05 | 0.685219 |  | Tgfb3 | 1.49 | 0.002888 |
| Csf2 | -1.92 | 0.447417 |  | Tgfbr1 | -1.04 | 0.014898 |
| Csf3 | -1.77 | 0.141223 |  | Tgfbr2 | 1.07 | 0.211584 |
| Ctsk | -32.70 | 0.000016 |  | Tgfbr3 | 1.09 | 0.339255 |
| Dlx5 | 2.77 | 0.001122 |  | Tnf | -3.08 | 0.070035 |
| Egf | 1.20 | 0.081415 |  | Tnfsf11 | -1.26 | 0.022793 |
| Fgf1 | -3.90 | 0.007061 |  | Twist1 | 1.06 | 0.207862 |
| Fgf2 | 1.39 | 0.004322 |  | Vcam1 | 1.26 | 0.038628 |
| Fgfr1 | -1.73 | 0.000710 |  | Vdr | 1.10 | 0.380209 |
| Fgfr2 | -1.73 | 0.001542 |  | Vegfa | -1.05 | 0.543994 |
| Flt1 | -1.23 | 0.082999 |  | Vegfb | -1.37 | 0.003804 |
| Fn1 | -1.34 | 0.013642 |  | Actb | 1.36 | 0.001746 |
| Gdf10 | 28.82 | 0.000750 |  | B2m | -1.06 | 0.377634 |
| Gli1 | 1.82 | 0.078176 |  | Gapdh | 1.97 | 0.000005 |
| Icam1 | -1.11 | 0.400958 |  | Gusb | -1.39 | 0.000837 |
| Igf1 | -1.64 | 0.000698 |  | Hsp90ab1 | -1.03 | 0.104054 |

**Supplement 3**. The list of genes analysed by Mouse Osteogenesis PCR Array. The columns show gene name, fold regulation and *P*-value.
