# Supplementary material for: Caspase-8 Deficient Osteoblastic Cells Display Alterations in Non-Apoptotic Pathways
Source: Front Cell Dev Biol. 2022 Mar 15;10:794407. doi: 10.3389/fcell.2022.794407 (PMC8964645; doi:10.3389/fcell.2022.794407)
Supplement: Supplementary file 2 [file DataSheet2.DOCX]

| **gene** | **fold reg.** | **P-value** |  | Gabarapl2 | 1.20 | 0.001283 |
| --- | --- | --- | --- | --- | --- | --- |
| Akt1 | 1.02 | 0.532819 |  | Hdac1 | 1.21 | 0.000451 |
| Ambra1 | 1.02 | 0.246937 |  | Hdac6 | 1.30 | 0.000143 |
| App | -1.07 | 0.076885 |  | Hgs | 1.01 | 0.665762 |
| Atg10 | -1.34 | 0.158434 |  | Hsp90aa1 | 1.25 | 0.002347 |
| Atg12 | 1.33 | 0.005394 |  | Hspa8 | -1.04 | 0.184340 |
| Atg16l1 | 1.07 | 0.337897 |  | Htt | 1.10 | 0.177458 |
| Atg16l2 | 1.11 | 0.193612 |  | Ifng | -1.26 | 0.333321 |
| Atg3 | 1.17 | 0.001536 |  | Igf1 | -5.87 | 0.000000 |
| Atg4a | 1.14 | 0.020909 |  | Ins2 | -1.01 | 0.767813 |
| Atg4b | 1.09 | 0.061512 |  | Irgm1 | 1.57 | 0.000272 |
| Atg4c | -1.05 | 0.356778 |  | Lamp1 | 1.11 | 0.001596 |
| Atg4d | 1.30 | 0.001500 |  | Map1lc3a | 1.42 | 0.000066 |
| Atg5 | 1.36 | 0.000024 |  | Map1lc3b | 1.02 | 0.453757 |
| Atg7 | 1.11 | 0.019805 |  | Mapk14 | 1.22 | 0.001245 |
| Atg9a | 1.19 | 0.001014 |  | Mapk8 | 1.18 | 0.011400 |
| Atg9b | 1.45 | 0.319511 |  | Mtor | 1.23 | 0.018384 |
| Bad | -1.10 | 0.009806 |  | Nfkb1 | 1.00 | 0.941401 |
| Bak1 | 1.34 | 0.000160 |  | Npc1 | -1.07 | 0.182507 |
| Bax | 1.23 | 0.000922 |  | Pik3c3 | 1.13 | 0.001172 |
| Bcl2 | 1.27 | 0.022013 |  | Pik3cg | 1.12 | 0.548388 |
| Bcl2l1 | 1.08 | 0.001096 |  | Pik3r4 | -1.04 | 0.470350 |
| Becn1 | 1.06 | 0.025645 |  | Prkaa1 | 1.17 | 0.001523 |
| Bid | 1.50 | 0.000003 |  | Pten | -1.01 | 0.752285 |
| Bnip3 | -1.07 | 0.005646 |  | Rab24 | 1.12 | 0.042809 |
| Casp3 | 1.53 | 0.000038 |  | Rb1 | 1.03 | 0.575003 |
| Casp8 | -1.28 | 0.000097 |  | Rgs19 | -1.10 | 0.058781 |
| Cdkn1b | -1.20 | 0.000099 |  | Rps6kb1 | 1.07 | 0.001637 |
| Cdkn2a | -1.01 | 0.767813 |  | Snca | -1.01 | 0.767813 |
| Cln3 | 1.05 | 0.882727 |  | Sqstm1 | 1.06 | 0.048464 |
| Ctsb | -1.30 | 0.010479 |  | Tgfb1 | 1.13 | 0.014683 |
| Ctsd | -1.08 | 0.000130 |  | Tgm2 | 1.51 | 0.003036 |
| Ctss | 1.55 | 0.372933 |  | Tmem74 | 1.32 | 0.021351 |
| Cxcr4 | -6.29 | 0.000207 |  | Tnf | 1.15 | 0.468138 |
| Dapk1 | -3.14 | 0.047789 |  | Tnfsf10 | -11.58 | 0.000994 |
| Dram1 | 1.02 | 0.518875 |  | Trp53 | 1.04 | 0.334033 |
| Dram2 | 1.13 | 0.015343 |  | Ulk1 | -1.07 | 0.074437 |
| Eif2ak3 | 1.26 | 0.001973 |  | Ulk2 | 1.06 | 0.096639 |
| Eif4g1 | 1.23 | 0.002712 |  | Uvrag | 1.04 | 0.385207 |
| Esr1 | 1.55 | 0.000456 |  | Wipi1 | -1.18 | 0.001470 |
| Fadd | 1.18 | 0.003665 |  | Actb | 1.21 | 0.007234 |
| Fas | 1.16 | 0.023236 |  | B2m | -1.14 | 0.020167 |
| Gaa | -1.41 | 0.001679 |  | Gapdh | -1.11 | 0.007402 |
| Gabarap | 1.06 | 0.017778 |  | Gusb | -1.10 | 0.153532 |
| Gabarapl1 | -1.01 | 0.798863 |  | Hsp90ab1 | 1.15 | 0.027126 |

**Supplement 2.** The list of genes analysed by Mouse Autophagy PCR Array. The columns show gene name, fold regulation and *P*-value.
