# Supplementary material for: Caspase-8 Deficient Osteoblastic Cells Display Alterations in Non-Apoptotic Pathways
Source: Front Cell Dev Biol. 2022 Mar 15;10:794407. doi: 10.3389/fcell.2022.794407 (PMC8964645; doi:10.3389/fcell.2022.794407)
Supplement: Supplementary file 3 [file DataSheet1.DOCX]

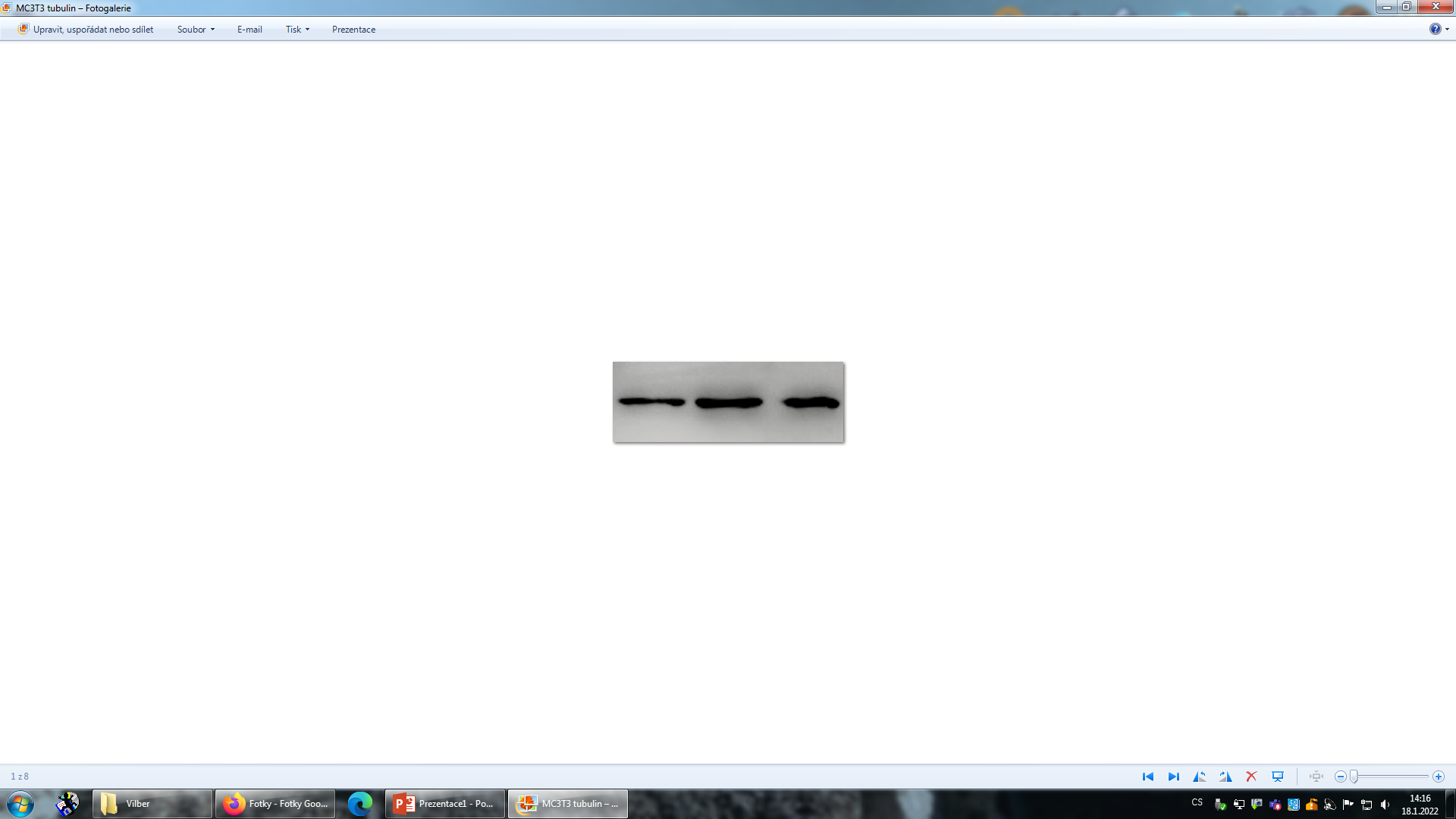

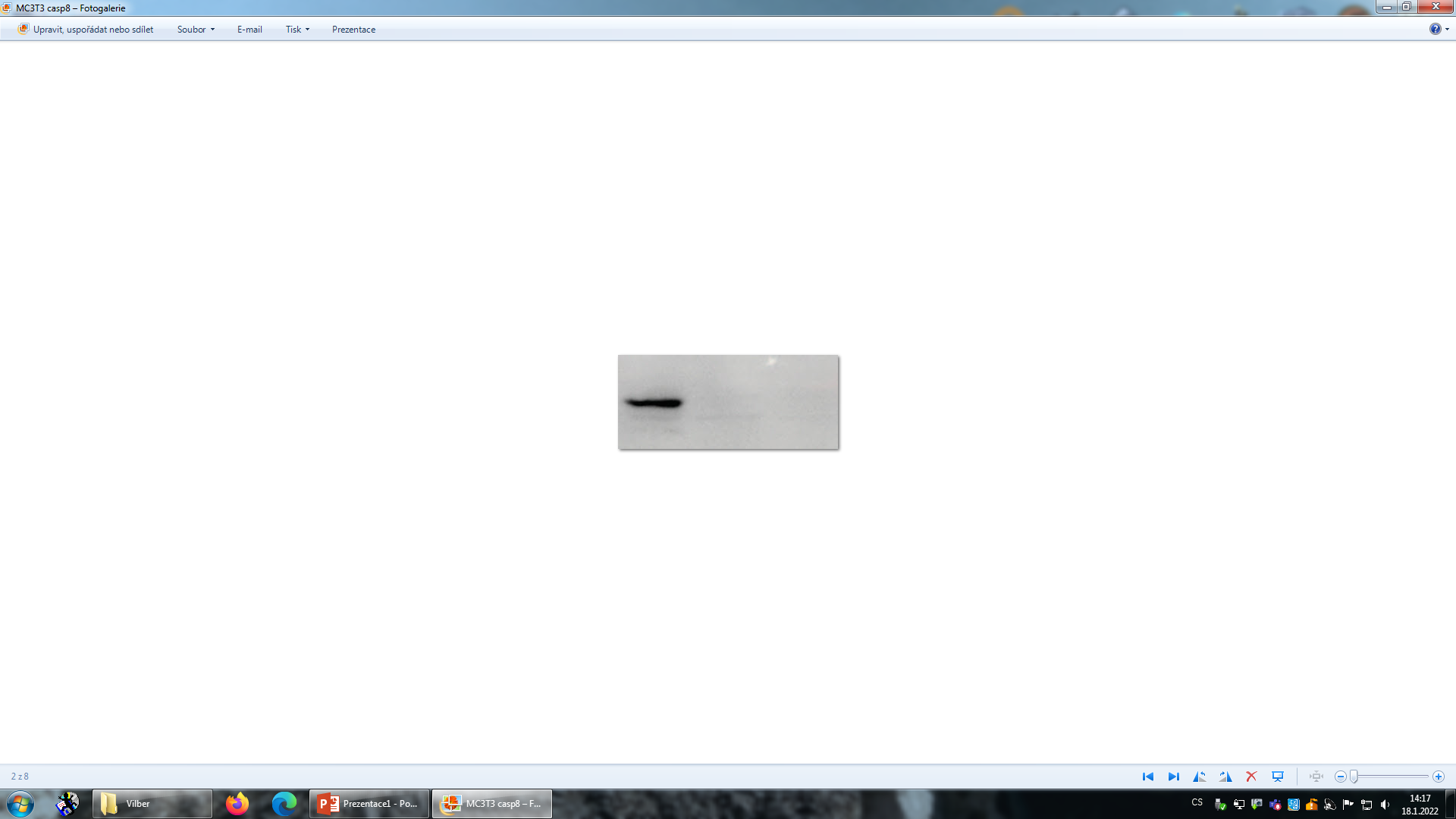


57 kDa

**caspase-8**

**full form**

**α-tubulin**

55 kDa

Ctrl

Casp8^-/- 2^


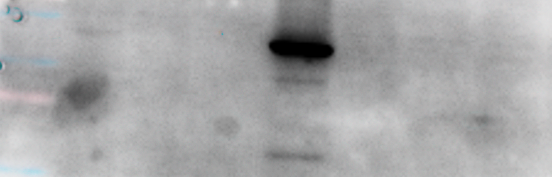


25 kDa -

35 kDa -

15 kDa -

55 kDa -

Doxorubicin

Untreated

cleaved caspase-8

α-tubulin

Ctrl

Casp8^-/-2^

**B**

55 kDa -


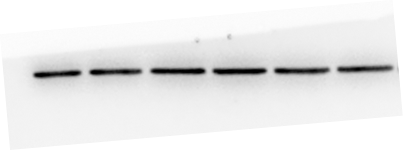


Casp8^-/-^

Ctrl

Casp8^-/-^

Casp8^-/-2^

**A**


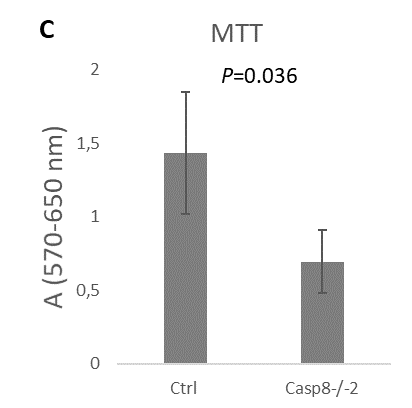


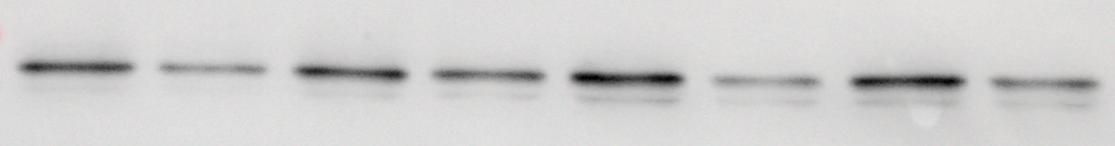

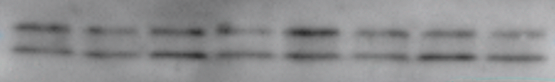


61 kDa -

Beclin-1

LC3B

16 kDa -

14 kDa -

Rapamycin

Rapamycin

α-tubulin

Ctrl

Ctrl

Casp8-/-

Casp8-/-

55 kDa -

**D**


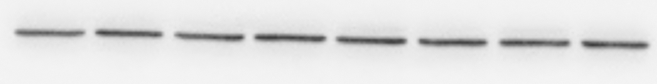

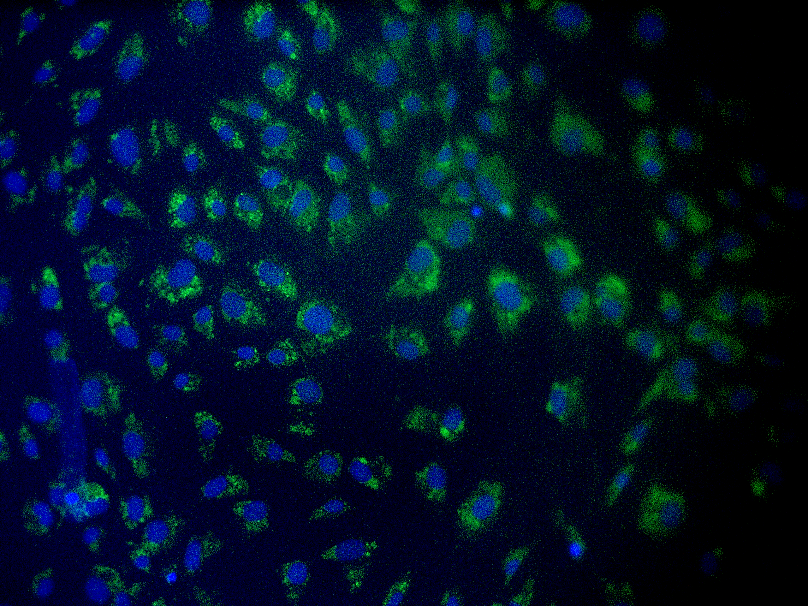

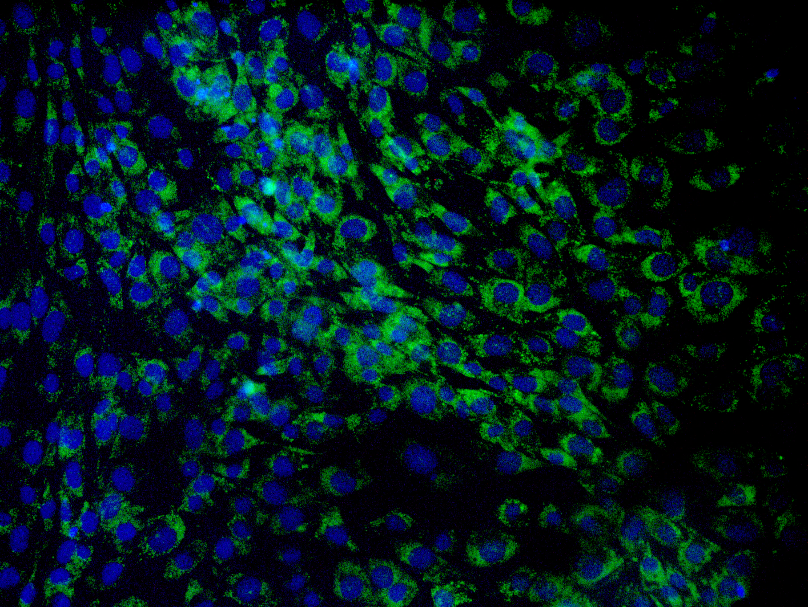

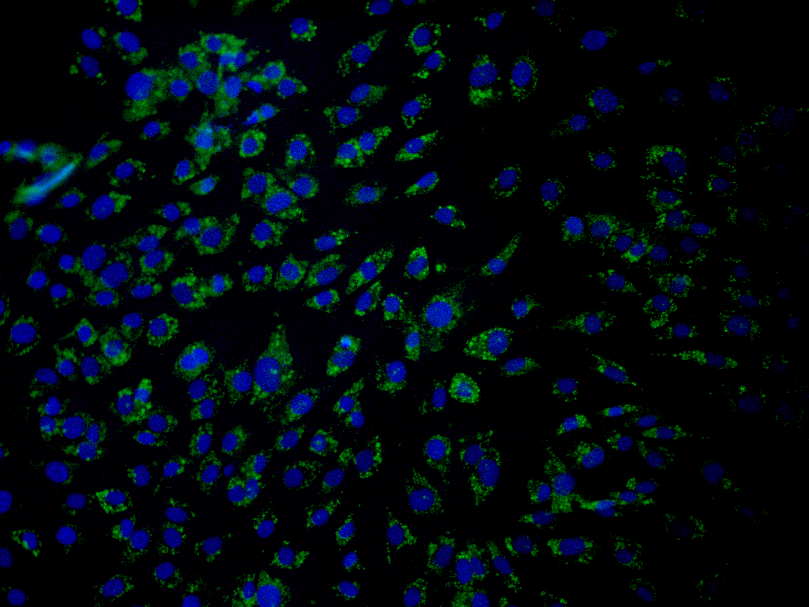

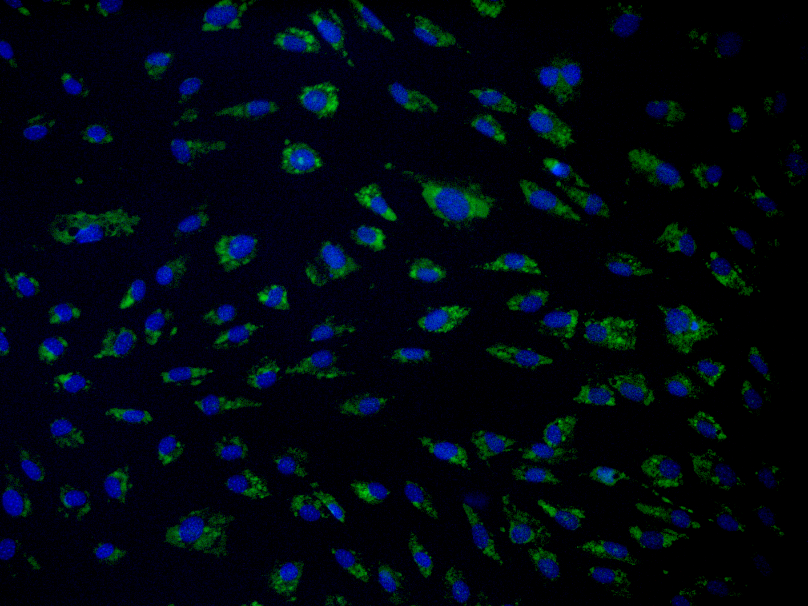


**E**

**H**

**G**

**F**

**Ctrl**

**Casp8^-/- 2^**

**Ctrl**

**Casp8^-/- 2^**

**Rap LC3B**

**Rap LC3B**

**UT LC3B**

**UT LC3B**

**Supplement 1.** Immunoblotting of full length caspase-8 proenzyme in unstimulated *Casp8^-/-2^* (clone 2) and control cells (A) and cleaved caspase-8 in doxorubicin treated and untreated cells (B). Control cells and two different *Casp8^-/-^* clones were analysed. Proliferation of *Casp8^-/-2^* cells compared to control cells evaluated by MTT test (C). Decreased proliferation was confirmed on another *Casp8^-/-^* clone (clone 2). Immunoblotting of Beclin-1 and LC3B in rapamycin treated *Casp8^-/-^* and control cells compared to untreated cells (D). Staining of LC3B (green) in artificially accumulated autophagosomes in control and *Casp8^-/-2^* cells after rapamycin treatment compared to untreated controls (E-H). Nuclei counterstained by DAPI (blue). Autophagosomes accumulation induced by chloroquine. Scale bar = 100 µm. **Rap** rapamycin, **UT** untreated
